# Enhancing Smile Aesthetics and Function with Lithium Disilicate Veneers: A Brief Review and Case Study

**DOI:** 10.3390/clinpract15030066

**Published:** 2025-03-18

**Authors:** Jose Villalobos-Tinoco, Franciele Floriani, Silvia Rojas-Rueda, Salwa Mekled, Clint Conner, Staley Colvert, Carlos A. Jurado

**Affiliations:** 1 Postgraduate Program in Periodontology and Implant Dentistry, National University of Rosario School of Dentistry, Rosario 3160, Argentina; 2 Independent Researcher and Clinician, Culiacan 80030, Sinaloa, Mexico; 3 Department of Prosthodontics, The University of Iowa College of Dentistry and Dental Clinics, Iowa City, IA 52242, USA; 4 Division of Dental Biomaterials, The University of Alabama at Birmingham School of Dentistry, Birmingham, AL 35233, USA; 5 Department of Restorative Dentistry, Temple University Kornberg School of Dentistry, Philadelphia, PA 19140, USA; 6 Division of Operative Dentistry, Department of General Dentistry, The University of Tennessee Health Science Center College of Dentistry, Memphis, TN 38104, USA; 7 Department of General Dentistry, The University of Tennessee Health Science Center College of Dentistry, Memphis, TN 38104, USA; 8 School of Dental Medicine, Ponce Health Sciences University, Ponce 00716, Puerto Rico

**Keywords:** lithium disilicate, veneers, aesthetic dentistry, smile

## Abstract

*Background*: Lithium disilicate ceramic veneers are considered the gold standard in aesthetic dentistry due to their translucency, strength, and adhesive bonding properties. This clinical case report details the aesthetic rehabilitation of a patient through the use of pressed lithium disilicate veneers, highlighting the treatment workflow, material selection rationale, and the long-term functional and aesthetic outcomes achieved. *Methods*: A review was conducted to evaluate the long-term success of lithium disilicate. A case study is presented that involves a 32-year-old female patient with anterior tooth discoloration, minor morphological discrepancies, and a desire for smile enhancement. A conservative approach using pressed lithium disilicate was chosen to restore harmony and enhance natural aesthetics. The treatment involved minimally invasive tooth preparation, digital smile design, and adhesive cementation using a total-etch technique with light-cured resin cement. High-resolution intra-oral and extra-oral photographs documented the case, capturing the preoperative, preparation, and final restoration stages. These images highlight shade matching, margin adaptation, and smile transformation after veneering. *Results*: Postoperative evaluation showed excellent aesthetic outcomes, color integration, and marginal adaptation, with the patient expressing high satisfaction. The veneers exhibited optimal translucency and strength, ensuring long-term durability. A one-year follow-up revealed no debonding, marginal discoloration, or surface degradation, confirming the clinical reliability of lithium disilicate veneers. *Conclusions*: Lithium disilicate provides predictability, durability, and high aesthetic results, making it an ideal choice for minimally invasive smile enhancement. The use of photographic documentation emphasizes the importance of case planning, precise preparation, and adhesive bonding for successful outcomes. Future research should focus on long-term survival rates and complication prevention to further refine material selection and bonding protocols.

## 1. Introduction

The demand for minimally invasive aesthetic restorations has significantly increased in modern dentistry, driving the widespread use of ceramic laminate veneers [1]. Among the available ceramic materials, lithium disilicate (LDS) has emerged as a preferred choice due to its superior mechanical strength, optical properties, and long-term clinical reliability [2]. Lithium disilicate veneers offer a balance of high fracture resistance and natural translucency, making them particularly suitable for aesthetic rehabilitation in the anterior region [3]. Compared to traditional feldspathic ceramics, lithium disilicate provides enhanced durability while maintaining a lifelike appearance, making it an excellent option for cases requiring color correction, shape modification, or minor alignment adjustments [4].

Clinical studies have demonstrated high survival rates and minimal complication rates associated with lithium disilicate veneers [5]. The long-term success of these restorations is largely dependent on proper case selection, preparation protocols, and adhesive cementation techniques [6]. When bonded to enamel, lithium disilicate demonstrates exceptional adhesion and superior longevity, reinforcing the importance of conservative tooth preparation to maximize bonding effectiveness [7]. The fabrication process of pressed lithium disilicate veneers further enhances their clinical performance. Using the lost-wax pressing technique, these restorations exhibit excellent marginal adaptation, high strength, and predictable aesthetic outcomes [8]. This technique allows for thin restorations with precise anatomical details, preserving the natural tooth structure while ensuring structural integrity [9]. Additionally, advances in adhesive cementation have improved the integration and longevity of lithium disilicate veneers, making them a reliable option for both functional and aesthetic rehabilitations [10].

This clinical case report details the aesthetic rehabilitation of a patient through the use of pressed lithium disilicate veneers, highlighting the treatment workflow, the material selection rationale, and the long-term functional and aesthetic outcomes achieved. The case underscores the effectiveness of minimally invasive preparation techniques, precise adhesive bonding protocols, and comprehensive post-treatment evaluation. By presenting these elements, the report demonstrates how lithium disilicate veneers can deliver natural-looking, durable, and highly aesthetic anterior restorations. The hypothesis of the study is that lithium disilicate can provide high aesthetic results that meet both the aesthetic and functional demands of patients. Additionally, the aim of the review is to examine the long-term success of previous studies evaluating lithium disilicate restorations in the aesthetic zone.

## 2. Materials and Methods

### 2.1. Literature Review

#### 2.1.1. Evolution of Lithium Disilicate Ceramics in Prosthodontics

Lithium disilicate (LDS) has emerged as one of the most widely used materials in aesthetic dentistry due to its superior mechanical properties, translucency, and adhesive bonding capabilities. Initially introduced as a pressable ceramic, LDS has undergone significant advancements in processing methods, leading to improvements in both its mechanical strength and clinical applications [11]. Its high flexural strength, ranging between 350–400 MPa, allows its use in monolithic restorations, providing a balance between aesthetics and function [12]. Compared to traditional feldspathic ceramics, LDS demonstrates enhanced resistance to fracture, which has led to its widespread adoption in minimally invasive prosthodontic procedures, such as veneers, inlays, onlays, and full-coverage crowns [13].

The studies included in the analysis were selected based on predefined inclusion and exclusion criteria to ensure relevance and quality. The inclusion criteria allowed studies that assessed lithium disilicate veneers for both anterior and posterior crowns, with a focus on survival rates, aesthetic outcomes, and mechanical performance. Only studies that were published in peer-reviewed journals and had both a clear methodology (prospective or retrospective) and a follow-up period of at least one year were included (Table 1). Additionally, the studies had to consider restoration fabrication methods, specifically whether the veneers were fabricated using digital or conventional techniques. The exclusion criteria eliminated studies with insufficient data on survival rates or aesthetic performance, studies involving restorations other than lithium disilicate, and those with follow-up periods shorter than one year. Studies not conducted on human subjects or those lacking rigorous control for variables such as patient age, health conditions, and occlusal loading were also excluded.

The analysis of the studies including anterior and posterior crowns assessed survival rates, aesthetic outcomes, and mechanical performance through retrospective and prospective methodologies. Additionally, the assessment considered whether restorations were fabricated using digital or conventional techniques (Table 2).

#### 2.1.2. Influence of Ceramic Thickness and Substrate on Clinical Success

The performance of lithium disilicate restorations is influenced by various factors, including ceramic thickness, bonding substrate, and occlusal loading conditions [11]. Research indicates that thinner restorations (≤1 mm) may exhibit comparable survival rates to standard-thickness restorations when adhesively bonded to enamel, emphasizing the role of proper adhesive protocols [12]. In an in vitro fatigue study, non-retentive lithium disilicate occlusal veneers (0.5 mm) demonstrated failure loads exceeding physiological chewing forces, suggesting their viability as a minimally invasive option [15]. Additionally, modified preparation designs, such as enamel-based occlusal veneers, have shown enhanced mechanical performance compared to conventional full-coverage crowns [17].

#### 2.1.3. Complications and Limitations of Lithium Disilicate Restorations

Despite its numerous advantages, lithium disilicate is not devoid of complications (Table 3). Fracture remains the most commonly reported failure, particularly in posterior restorations under heavy occlusal loads [18]. The susceptibility of thin LDS restorations to bulk fractures underscores the importance of appropriate case selection and occlusal adjustment to mitigate excessive stress concentration [19]. Additionally, issues such as chipping and marginal degradation have been observed in some cases, necessitating periodic maintenance and follow-up appointments [20]. While recent studies have highlighted the success of LDS in implant-supported prostheses, the lack of long-term clinical data remains a limitation, calling for further research to evaluate its performance over extended treatment durations.

#### 2.1.4. Future Perspectives on Lithium Disilicate Applications

The ongoing advancements in digital dentistry and CAD/CAM technology continue to refine the application of lithium disilicate in prosthodontics (Table 4). The integration of fully crystallized LDS blocks for chairside fabrication has improved workflow efficiency while maintaining comparable mechanical and aesthetic outcomes [21]. Future research should focus on optimizing material properties to enhance longevity, particularly for implant-supported prostheses and minimally invasive restorations [22]. Additionally, further clinical trials comparing LDS with alternative ceramic systems, such as zirconia-reinforced lithium silicate, may provide valuable insights into material selection for various clinical scenarios.

### 2.2. Case Report

**Figure 1:**
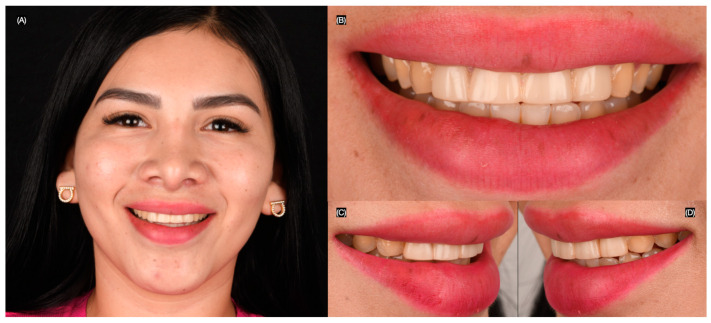
Initial extra-oral. (**A**) Face smiling; (**B**) frontal, (**C**) right side, and (**D**) left side views of smile.

**Figure 2:**
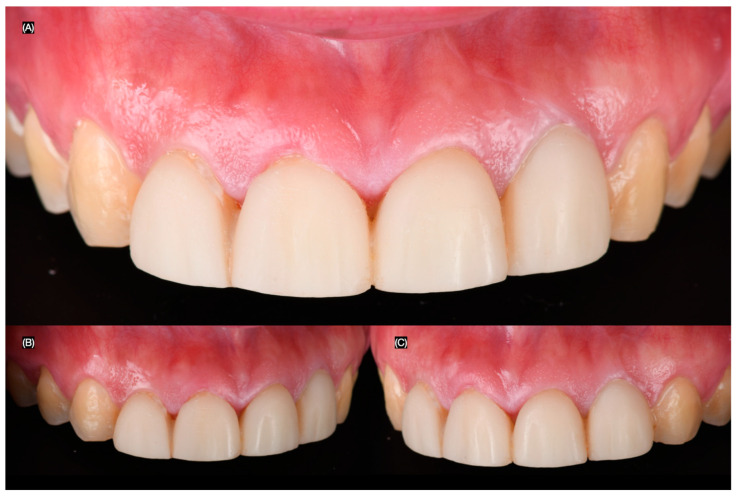
Initial intra-oral. (**A**) Frontal, (**B**) right side, and (**C**) left side views.

A 25-year-old female patient presented with the chief complaint of disliking her smile. She stated that she had previously received large composite direct restorations from the right lateral incisor to the left lateral incisor. Upon clinical evaluation, the following issues were diagnosed: stained resin composite restorations from the right lateral incisor to the left lateral incisor, incisal wear from canine to canine, and non-ideal gingival zenith levels from canine to canine (Figure 1 and Figure 2). This patient was selected for the case study due to the presence of anterior tooth discoloration, minor morphological discrepancies, and a desire for smile enhancement, which made her a suitable candidate for minimally invasive treatment with lithium disilicate veneers. Similar cases were reviewed, but this patient’s specific clinical needs, including her preference for a conservative approach, made her the ideal candidate for the procedure.

The patient was presented with different treatment options, including crown lengthening to improve the gingival architecture of the anterior dentition, tooth whitening, and replacement of the four resin composite restorations with all-ceramic restorations. Additionally, due to her wide smile, veneer restorations were proposed from the right second premolar to the left second premolar. The patient disliked the idea of crown lengthening and was informed that, because of her low smile, the treatment could be completed without it. She also disliked tooth whitening, as she claimed to have previously undergone the procedure without any improvement.

**Figure 3:**
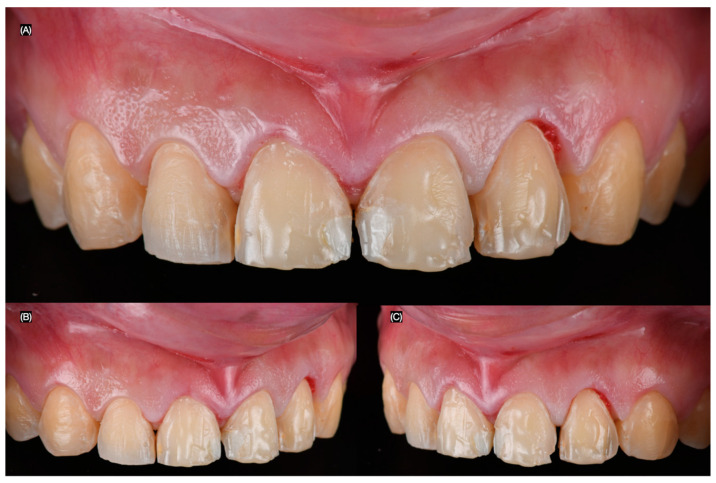
Partial removal of resin composite restorations from right to left lateral incisor. (**A**) Frontal, (**B**) left side, and (**C**) right side views.

**Figure 4:**
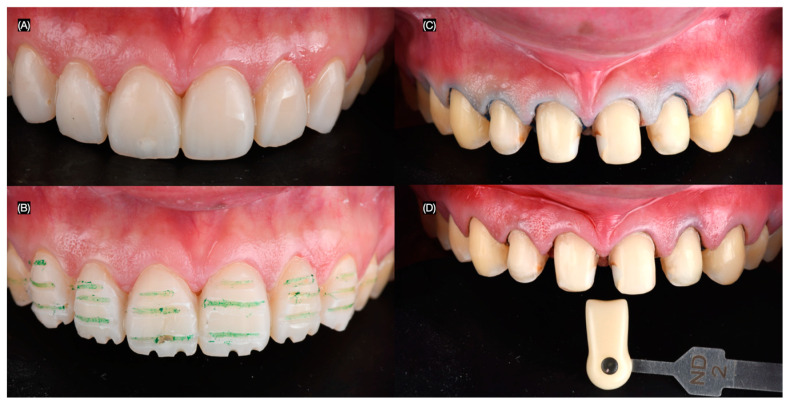
Mock-up and preparation. (**A**) Intra-oral mock-up, (**B**) tooth preparation on mock-up, (**C**) final tooth preparations with cord packed, and (**D**) photo with shade guide for ceramic restoration fabrication.

The patient was interested in minimally invasive veneer restorations for all anterior teeth visible in her wide smile and requested to proceed with the treatment. A diagnostic wax-up was created, followed by an intra-oral mock-up to assess the proposed shape of the restorations. The patient approved the shade and agreed to continue with the treatment. The resin composite restorations on the four anterior teeth were partially removed, and the patient was informed that, due to the large size of the restorations, these teeth would require full-coverage crowns, while the rest would receive veneers (Figure 3). The depth of reduction for the veneers was carefully planned to preserve as much healthy tooth structure as possible while ensuring proper adaptation of the veneers. A minimal reduction approach was utilized, with a depth of approximately 0.5 mm at the incisal edge and the cervical area, allowing for optimal aesthetic results while maintaining structural integrity. The margin design was a chamfer type, which was selected for its ability to provide a smooth, well-defined edge that allows for strong adhesive bonding and a seamless transition between the veneer and the natural tooth structure (Figure 4).

**Figure 5:**
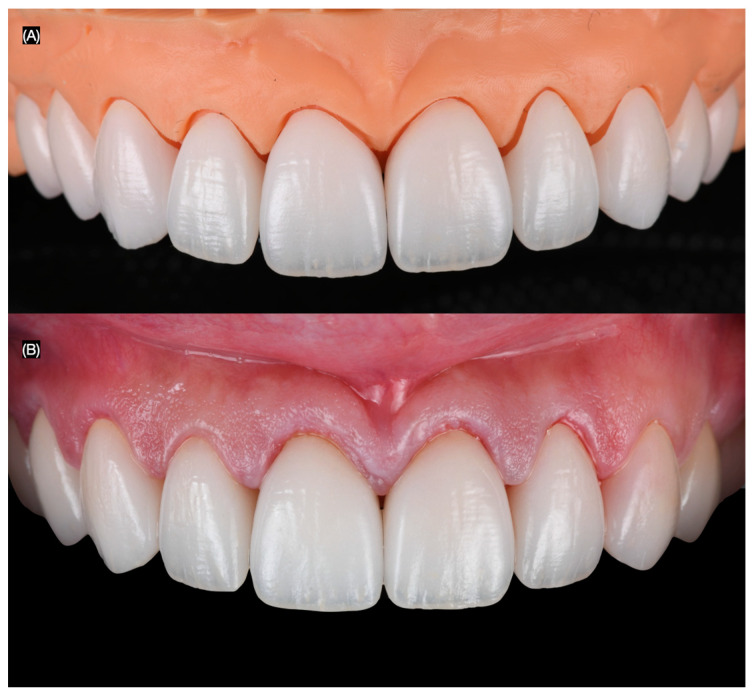
Lithium disilicate restorations. (**A**) Restorations in master model and (**B**) intra-oral try-in.

Ten hand-crafted lithium disilicate veneer restorations were fabricated spanning from the right second premolar to the left second premolar. A dry try-in of the restorations was performed to evaluate the margins, contours, and shade. The patient approved the restorations and requested to proceed with the cementation procedure (Figure 5).

**Figure 6:**
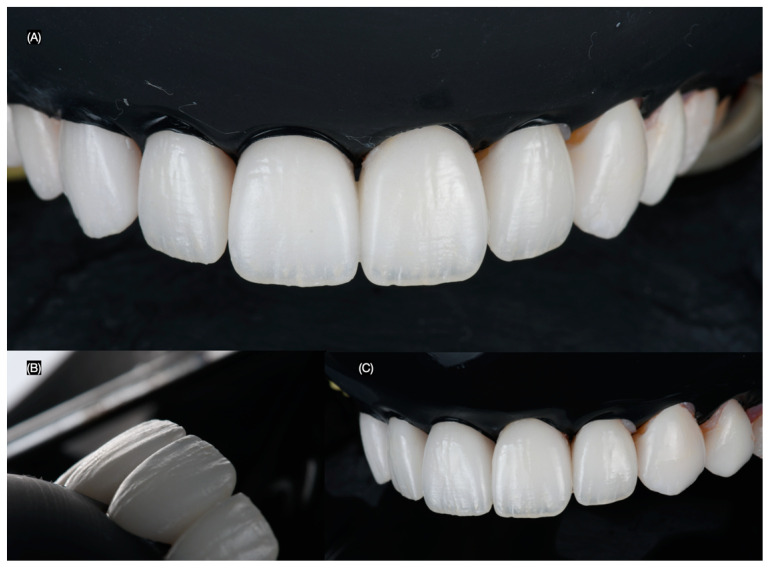
Dental isolation during bonding protocol. (**A**) Frontal, (**B**) lateral, and (**C**) left side views.

Total isolation was achieved with a black dental dam, spanning from the right first molar to the left first molar. The restorations were first treated with hydrofluoric acid (Porcelain Etch, Ultradent, South Jordan, UT, USA) for 20 s, then rinsed and dried. Next, the restorations were treated with a silane coupling agent (Monobond Plus, Ivoclar Group, Schaan, Liechtenstein) for 60 s and air-dried. The teeth were prepared by first using 29-micron aluminum oxide particles and water (AquaCare, Velopex, London, UK), followed by rinsing and air-drying. Then, 37% phosphoric acid (Total Etch, Ivoclar Group, Schaan, Liechtenstein) was applied for 15 s, and the teeth were air-dried. Lastly, an adhesive was applied, and the restorations were cemented with resin cement (Variolink Esthetic LC, Ivoclar Group, Schaan, Liechtenstein). Each surface (facial, mesial, distal, and incisal) was light-cured for 20 s. Excess cement was removed with a #12 blade (Figure 6).

**Figure 7:**
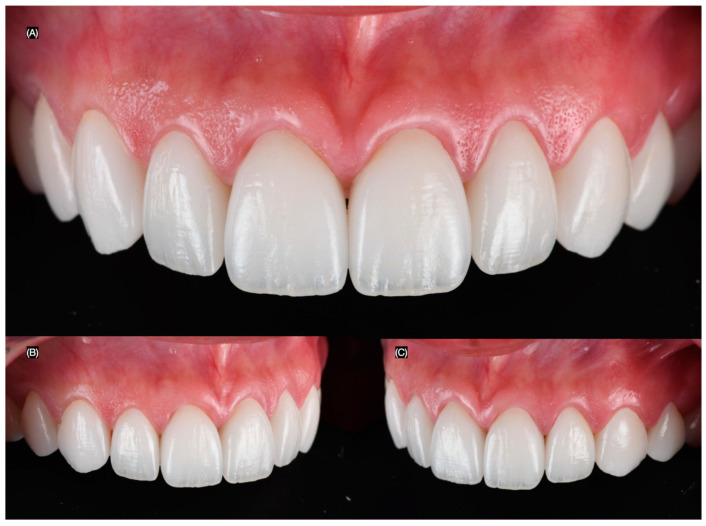
Final intra-oral. (**A**) Frontal, (**B**) right side, and (**C**) left side views.

**Figure 8:**
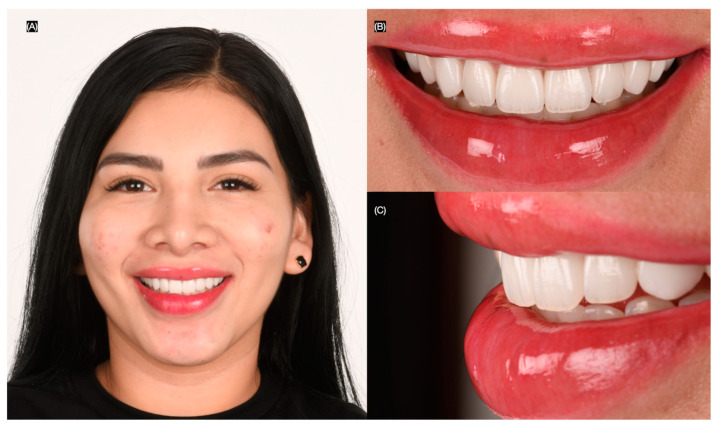
Final extra-oral. (**A**) Face smiling; (**B**) frontal and (**C**) left side views of smile.

The patient was pleased with the final outcome and was provided with an occlusal guard to wear at night to protect the restorations (Figure 7 and Figure 8).

## 3. Results

The hypothesis was confirmed because the lithium disilicate veneer restorations in the aesthetic zone met the patient’s aesthetic and functional demands. Furthermore, the literature review we conducted showed that lithium disilicate restorations in the aesthetic zone can provide predictable and positive long-term results. During the clinical case, lithium disilicate veneers demonstrated highly aesthetic and functional outcomes. Postoperative evaluation indicated excellent color matching, natural translucency, and smooth integration with the surrounding dentition. The patient exhibited high satisfaction with the final smile design, reporting an improvement in self-confidence and overall aesthetic perception.

Marginal adaptation was evaluated both clinically and through high-resolution photography, confirming the precise fit of the veneers without visible gaps or overcontoured areas. The adhesive bonding technique using the total-etch protocol and light-cured resin cement contributed to the strong adhesion and seamless integration of the restorations.

**Figure 9:**
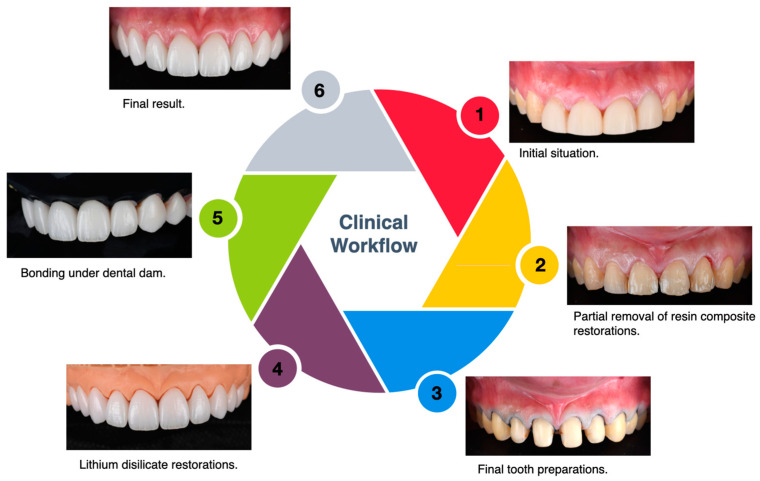
Summary of case study workflow.

Follow-up assessments at three months, six months, and one year confirmed the absence of complications such as debonding, marginal discoloration, chipping, or wear. The patient maintained proper oral hygiene, and no gingival inflammation or secondary caries were observed. These findings support the clinical reliability and long-term stability of lithium disilicate veneers in aesthetic rehabilitations. A flowchart describing the steps of the clinical workflow implemented can be seen in Figure 9.

## 4. Discussion

The success of ceramic veneers in aesthetic dentistry depends on multiple factors, including material selection, preparation design, adhesive cementation, and long-term clinical performance. Among the available ceramics, lithium disilicate (LDS) has demonstrated superior mechanical properties, high survival rates, and lower complication rates, making it a widely preferred choice for aesthetic rehabilitations. The findings from Klein et al. (2024) further reinforce the reliability of lithium disilicate, reporting a 96.81% survival rate over 10.4 years, significantly outperforming feldspathic and leucite-reinforced ceramics [1]. The present case illustrates the effective use of pressed lithium disilicate veneers in achieving functional and aesthetic success, aligning with the current literature on minimally invasive ceramic restorations.

A key factor contributing to the longevity of lithium disilicate veneers is adhesive bonding to enamel, which enhances fracture resistance and retention. Studies have shown that bonding lithium disilicate to enamel significantly reduces the risk of debonding and marginal discoloration, particularly when using etch-and-rinse adhesive systems [22]. In the present case, a conservative preparation approach was utilized to maintain the maximum amount of enamel surface for bonding, ensuring optimal adhesive performance. This is consistent with clinical recommendations emphasizing minimized dentin exposure to enhance the long-term stability of bonded restorations [23].

When compared to alternative ceramic materials, lithium disilicate offers superior fracture resistance due to its high flexural strength (360–400 MPa), making it less prone to chipping than feldspathic ceramics [24]. Additionally, aesthetic complications such as discoloration and surface roughness are less frequent in lithium disilicate veneers compared to feldspathic and zirconia-based restorations. The present case confirms this trend, as the patient exhibited high aesthetic satisfaction with shade matching and surface gloss, with no visible marginal discoloration post-treatment.

Despite their advantages, lithium disilicate veneers can still be susceptible to technical failures, particularly in cases of parafunctional habits or occlusal overload [25]. The present case incorporated protective measures such as an occlusal splint to mitigate excessive forces, a strategy supported by previous studies to enhance veneer longevity [26]. In comparison to other ceramic materials, lithium disilicate veneers offer superior aesthetics, mechanical properties, and longevity [27]. While zirconia is known for its high fracture resistance and durability, it lacks the translucency that lithium disilicate provides, which is crucial for achieving a natural aesthetic appearance, particularly in the anterior region. Feldspathic ceramics, on the other hand, are highly aesthetic and have been used in veneers for many years; however, they tend to be more prone to chipping and fracture under heavy occlusal forces [28]. Lithium disilicate strikes an excellent balance between strength and aesthetics, with a flexural strength of 350–400 MPa, making it more durable than feldspathic ceramics and offering better wear resistance compared to zirconia in some cases [29]. Furthermore, the long-term survival rate of lithium disilicate is supported by clinical studies that highlight its decreased marginal discoloration and chipping compared to feldspathic ceramics, making it an ideal choice for minimally invasive restorative procedures [30].

A limitation of this study is that it is based on a single case, which may not fully represent the potential variability in outcomes that could arise from different patient conditions. Patient-specific factors such as occlusion, oral hygiene, and parafunctional habits may influence the long-term success of lithium disilicate veneers, and further studies involving larger sample sizes and diverse patient populations are needed to confirm these findings. Moreover, periodic follow-up evaluations were scheduled to monitor restoration integrity and assess potential wear. These steps align with the best practices recommended in the literature to reduce mechanical complications and enhance clinical outcomes [31].

## 5. Conclusions

The findings from this case, along with supporting evidence from recent studies, reaffirm the predictable success of pressed lithium disilicate veneers when proper case selection, preparation protocols, and adhesive bonding techniques are carried out. The one-year follow-up confirmed the stability of the restorations, with no evidence of debonding, marginal discoloration, or fractures, reinforcing the long-term reliability of lithium disilicate in aesthetic rehabilitations.

Overall, this case highlights the importance of material selection, proper bonding techniques, and ongoing patient follow-up in achieving long-term aesthetic and functional success with lithium disilicate veneers. Future research should explore the long-term performance of lithium disilicate veneers under different clinical conditions, including varying occlusal loads, preparation depths, and cementation protocols, to further refine clinical guidelines for optimizing their success. Advances in CAD/CAM technology and adhesive techniques will continue to enhance treatment protocols, optimizing minimally invasive approaches for improved aesthetics and function.

## Figures and Tables

**Table 1:** Inclusion and exclusion criteria used for studies included in review.

Inclusion Criteria	Exclusion Criteria
Lithium disilicate veneers	Lithium disilicate implant crowns
Lithium disilicate non-prep veneers	Full mouth reconstructions
Lithium disilicate crowns	Cases without survival rate information
Providing the fabrication methods	Cases not in the aesthetic zone
At least 1 year follow-up	No follow-up provided

**Table 2:** Survival rate by location of lithium disilicate.

Reference	Material Evaluated	Location	Fabrication Method	Evaluation Method
Smielak et al. (2022) [8]	Lithium Disilicate Veneers	Anterior	Conventional	Survival rate comparison over 9 years
Yıldırım et al. (2023) [14]	Lithium Disilicate Veneers	Anterior	Conventional	FDI criteria over 2 years
Peumans et al. (2004) [9]	Porcelain Veneers	Anterior	Conventional	10-year prospective clinical trial
Aslan et al. (2019) [10]	Pressable Glass–Ceramic Veneers	Anterior and Posterior	Conventional	Retrospective case series study (5, 10, 15, 20 years)
Aslan et al. (2019) [11]	Lithium Disilicate Laminate Veneers	Anterior	Conventional	10-year retrospective study
De Angelis et al. (2023) [12]	No-Prep Porcelain Veneers	Anterior	Conventional	Retrospective evaluation of clinical performance
Demirekin and Turkaslan (2022) [13]	Laminate Veneer Ceramics	Anterior	Conventional	10-year follow-up study on fluorosis cases
Fabbri et al. (2014) [15]	Lithium Disilicate Restorations (Anterior and Posterior)	Anterior and Posterior	Digital and Conventional	3-to-6-year retrospective study
Fradeani et al. (2005) [16]	Porcelain Laminate Veneers	Anterior	Conventional	6-to-12-year retrospective study
Gonzalez-Martin et al. (2021) [17]	Ultrathin Ceramic Veneers	Anterior	Digital	36-month retrospective case series
Imburgia et al. (2021) [18]	Lithium Disilicate CAD/CAM Veneers	Anterior	Digital	Feather-edge margins evaluation in 105 patients

**Table 3:** Clinical complications involving lithium disilicate dental material.

Material	Survival Rate (5)	Technical Complications (%)	Aesthetic Complications (%)	Biological Complications (%)	Follow-Up Period (Years)
Feldspathic Ceramic	88.2	15.6	4.8	3.9	8.2
Leucite-Reinforced Ceramic	91.5	12.4	3.2	2.8	9.0
Zirconia-Based Ceramic	94.3	8.7	2.5	1.2	9.8

**Table 4:** Studies evaluating the survival of dental ceramics.

Author(s) and Year	Study Focus	Methodology	Key Findings
Klein et al., 2024 [1]	Survival and complication rates of ceramic veneers	Systematic review and meta-analysis	Lithium disilicate veneers demonstrated a 96.81% survival rate over 10.4 years, outperforming feldspathic and leucite-reinforced ceramics.
Polat et al., 2024 [1]	Survival of polymer-infiltrated ceramic network restorations	Retrospective clinical study	PICN restorations had high survival rates up to three years but higher complications compared to lithium disilicate.
Gierthmuehlen et al., 2024 [3]	Effect of ceramic thickness on failure load of occlusal veneers	Laboratory fatigue study	Thin (1.0 mm) and ultrathin (0.5 mm) lithium disilicate veneers bonded to enamel showed superior mechanical performance.
Strasding et al., 2024 [4]	Material selection for implant-supported prostheses	Narrative review	Lithium disilicate single crowns in implant-supported restorations had a 3-year survival rate of 97.0%, comparable to zirconia.
Yli-Urpo et al., 2025 [5]	Cement thickness and load-bearing capacity	Laboratory study	Optimized cement layer thickness improved mechanical performance and reduced fracture risk in lithium disilicate restorations.
Margvelashvili-Malament et al., 2025 [6]	Minimally invasive prosthodontics	Narrative review	Lithium disilicate is preferred for minimally invasive restorations due to its high strength, aesthetics, and bonding properties.
Shoorgashti et al., 2024 [7]	Effect of surface treatments on lithium disilicate bonding	Systematic review	Surface treatments significantly influence the adhesive properties and longevity of lithium disilicate restorations.

## Data Availability

The data presented in this study are available on request from the corresponding author.
